# The Potential Role of Seminal Plasma in the Fertilization Outcomes

**DOI:** 10.1155/2019/5397804

**Published:** 2019-08-20

**Authors:** Justyna Szczykutowicz, Anna Kałuża, Maria Kaźmierowska-Niemczuk, Mirosława Ferens-Sieczkowska

**Affiliations:** ^1^Department of Chemistry and Immunochemistry, Wroclaw Medical University, Wroclaw, Poland; ^2^3rd Department and Clinic of Paediatrics, Immunology and Rheumatology of Developmental Age, Wroclaw Medical University, Wroclaw, Poland

## Abstract

For human infertility both male and female factors may be equally important. Searching for molecular biomarkers of male infertility, neglected for decades, and the attempts to explain regulatory mechanisms of fertilization become thus extremely important. Apart from examination of the structure and function of male gametes, also the possible importance of seminal plasma components should be considered. In this article we discuss data that indicate for the substantial significance of active seminal plasma components for conception and achievement of healthy pregnancy. Seminal plasma impact on the storage and cryopreservation of human and animal sperm and regulatory role of glycodelin on human sperm capacitation as well as hypothesized course of female immune response to allogenic sperm and conceptus has been discussed. The possible involvement of carbohydrates in molecular mechanism of fetoembryonic defense has been also mentioned.

## 1. Introduction

Decreased fertility is now becoming an important social and medical problem, especially in the industrial countries. Infertility must be taken into consideration when the couple cannot achieve pregnancy after one year of regular unprotected intercourse. Such problems affect 15-20% of couples, as estimated by WHO [1–4]. In 20-60% of infertility cases the male factor is at least coexistent. Apart from anatomical reasons such as cryptorchidism and varicocele, reduced male fertility used to be associated with abnormal semen parameters, including reduced sperm count, motility, and/or abnormal sperm morphology [5]. Today andrologists agree that the reference to normative semen parameters does not provide a physician with sufficient information on the reproductive potential of a particular patient [2, 4, 6–8]. On the one hand, male subjects whose semen do not fulfill all the WHO criteria can often become fathers without any serious problems, but also, males whose sperm meet WHO criteria may face problems with conceiving their offspring. Such cases are classified as unexplained male infertility (UMI) [9, 10]. The prevalence of UMI among infertile men is estimated for about 15%, although in some population studies the values of up to 37% have been reported [10].

Among the various reasons associated with male fertility problems, the most attention is devoted to the sperm disorders. The integrity of sperm DNA is of primary significance [4, 11–13]. Numerous recent studies underline also the importance of genetic polymorphism, as summarized recently in the paper by Havrylyuk et al. [14] Oxidative stress and excessive production of reactive oxygen species (ROS) [15–19] are known to negatively affect reproductive potential. High oxidative power is necessary for the appropriate sperm function. Reactive oxygen species are involved in sperm capacitation, hyperactive motion, and acrosomal reaction. However, too much ROS resulting from extrinsic exposure (environmental and industrial pollution and cigarette smoking) impairs the balance and leads to DNA damage, peroxidation of lipids in sperm, and mitochondrial membranes and increases apoptosis [20]. Except improvement of the lifestyle and, first of all, giving-up smoking, the male patients are often recommended oral antioxidant supplementation [12, 21–23] to improve the redox balance. Unfortunately, as dosing antioxidants is not currently regulated, the anxiety arises that overdosage of such compounds may inhibit physiological oxidation mechanism and may be finally detrimental for sperm function [20]. In our recent study, we found surprisingly reduced oxidative potential in the group of infertile men, possibly attributable to the excessive use of antioxidant supplements [24].

The incidence of DNA damage and redox imbalance as reasons of male infertility is estimated at around 10% for each of these factors in the total number of UMI cases [9, 10]. Thus for a large group of patients, the infertility background still remains unexplained. The factors less understood so far may comprise a molecular background of sperm activation and the interactions involved in the achievement of an appropriate maternal immune balance.

This article is aimed to bring the reader to some contemporary concepts concerning selected aspects of reduced male fertility, including the protection of sperm from premature capacitation, interaction with the cervical mucus, and interactions governed by female immune system. The molecular background of these cascades of events is still far from complete understanding.

## 2. Is Seminal Plasma Important for Fertilization?

Spermatozoa stored and transported within the male reproductive tract are initially accompanied by testicular and epididymal secretions. At the moment of ejaculation, male gametes from the epididymides mix with the secretions of accessory glands of the male reproductive tract. Components derived from testicles and epididymides, as well as from prostate, seminal vesicles and seminiferous tubule epithelium form seminal plasma (SP): a complex mixture of ions, organic compounds (e.g., citric acid), sugars (fructose), prostaglandins, and various proteins [25, 26]. Seminal plasma was for a long time considered a passive medium delivering sperm to the female reproductive tract; over a time, however, it began to be attributed to a greater functional role in effective reproduction [26, 27]. In addition to providing sperm with proper nutrition and protection from the harmful vaginal environment, seminal plasma components are responsible for regulation of capacitation, survival time of the gametes in female reproductive tract, and, last but not least, conditioning of the female immune system, elaborating the tolerance to allogenic embryos [28, 29].

After coitus the ejaculate is immediately coagulated in the partner's vagina. The two most abundant seminal plasma proteins, semenogelin and fibronectin, form a network of cross-linked fibers that cause gelation and immobilization of the sperm. This clot, deposited in the cervical os, is successively liquefied within about an hour from intercourse. Seminal plasma proteolytic enzymes cut the network of semenogelin and fibronectin polymers, gradually releasing the sperm. The primary protease involved in the process is prostate specific antigen (PSA), supported by metalloproteinases, mainly the members of the gelatinase group (MMP-9 and MMP-2) [24, 30]. The released spermatozoa are still not ready to fertilize the egg. They penetrate the plug of cervical mucus, getting into the uterus cavity. Then the capacitation becomes possible: a cascade of changes in the cell membrane that result in sperm hyperactive motion and gaining the ability to attach to oocyte* zona pellucida* (ZP). This attachment leads to acrosomal reaction, the final activation of male gametes, cleavage of the ZP glycoproteins, and fusion of the sperm and the oocyte. This in turn activates the mechanism preventing fusion of next male gametes with the oocyte (polyspermia blockade). Until recently it was thought that due to the short duration of seminal plasma interaction with sperm, and the fact that in humans SP components do not pass through the cervical mucus to the upper parts of the female reproductive tract, the contribution of seminal plasma components in the regulation of the fertilization process is limited. It is also well known that even immature spermatozoa directly extracted from epididymides are able to fertilize an egg under some conditions. The procedure is used in assisted reproduction techniques (ART), including in vitro fertilization (IVF) and intracellular sperm injection (ICSI) [26, 31, 32]. The ability to fertilize an egg presented by the gametes that has never been in contact with seminal plasma once again cast doubt on the demands of SP components for the successful fertilization. However, considering SP a passive medium is nowadays challenged by the results of a number of studies.

Supplementation with seminal plasma was shown to reduce the deleterious effect of cryopreservation in both human and animal sperm. Although sperm cells are relatively resistant to freezing, all the procedure comprising semen dilution, washing, freezing, and thawing leads to a considerable loss of sperm function. The recovery of vital human sperm occurred significantly higher when the sperm pellet was resuspended in autologous SP prior to freezing [33]. Ben et al. [34] showed that this positive effect is dependent on SP concentration, and Donnelly [35] proved that supplementation with SP diminished DNA-impairment and improved sperm motility during cryopreservation. Recent years have brought some interesting reports on the role of seminal plasma proteins in the effectiveness of fertilization in farm animals [36–41]. Since controlled breeding is widely used and economically viable practice in husbandry, the research focused on the phenomena accompanying fertilization has been intensified. Frozen and thawed ram sperm were more likely to recapture the ability to fertilize sheep when thawing was accompanied with addition of seminal plasma [36, 37]. The fertilization potential after cryopreservation was also modulated by differences in seminal plasma composition [39, 40]. The positive effect of seminal plasma on sperm condition has been also reported for boars and stallions, while in bulls detrimental effects prevailed [37, 42, 43]. The authors suggest that interspecies variability of seminal plasma composition may result in different regulatory effects.

Rickard et al. [39] reported that the exposition of epididymal spermatozoa to seminal plasma increased their ability to reach the uterus after insemination that was measured as a number of sperm reaching uterus cavity after a time. Interestingly, such “more efficient” gametes did not show any alterations in the kinetic parameters, motility, or mitochondrial membrane potential. The beneficial effect of SP was therefore suggested to be associated with sperm ability to penetrate cervical mucus. The molecular mechanisms of these phenomena have not been elucidated.

In humans and the other primates facilitation of sperm penetration through the cervical mucus has been attributed to *β*-defensin 126, a glycosylated polypeptide produced in the epididymides and adsorbed on the sperm surface [27, 44].

## 3. Factors Involved in Capacitation Control

**Figure 1:**
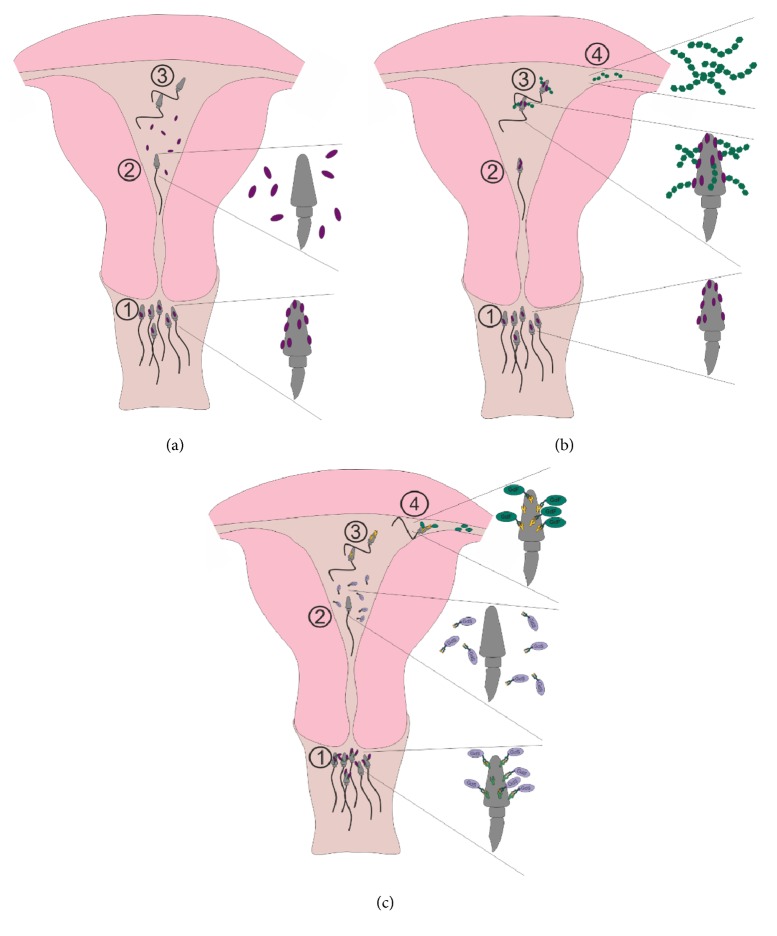
The concepts of involvement of seminal plasma factors in regulation of sperm capacitation (a): 1: sperm coated with SP decapacitation factors (BSP, binder of sperm proteins); 2: decapacitation factors shedded; 3: capacitated sperm: hyperactive motion (b): 1: sperm coated with decapacitation factors (BSP); 2: uterine cavity: BSP still on the sperm head; 3: attachment of GAGs: capacitation; 4: tubular junction GAGs: chemotaxy and capacitation signal (c): 1: sperm coated with SP glycodelin S (Gd-S); 2: Gd-S shedded: capacitation; 3: capacitated sperm; 4: attachment of Gd-F.

Spermatozoa have to get their full competence to recognize the oocyte neighbourhood and penetrate the cumulus cells area just at the moment when they reach the fallopian tube. This is a challenge demanding strict regulation of time and site activation of male gametes. Except semen gelation and gradual release of sperm during liquefaction, capacitation control is crucial. It can be initiated only when sperm traverse the cervical mucus and reach the uterus cavity. Seminal plasma factors responsible for stabilization of the cell membrane thus preventing too early capacitation are in general termed decapacitation factors [42, 45]. Data on these factors come mainly from the research on sperm handling for farm animals controlled breeding. Initially found in the bovine semen, now decapacitation factors are suggested to be conserved among the species and termed binder of sperm proteins (BSP) [42]. This is a family of small (12-30 kDa) proteins containing fibronectin type-2 domain. These proteins are tightly but temporarily adsorbed to the phosphatidylcholine lipids of sperm membrane. In the uterus cavity these factors are shedded from the sperm surface and this leads to cholesterol efflux, membrane destabilization, and remodeling, the first steps of activation of the male gametes (Figure 1(a)).

Some data suggest however that in bulls BSP binding to the sperm surface is stronger and more permanent than observed in rams and boars [42]. This feature may hinder capacitation and be responsible for detrimental rather than beneficial effect of SP supplementation in sperm handling in these animals. An interesting feature of BSPs is their ability to recognize heparin-like glycosaminoglycans (GAGs) and high density lipoproteins. Such compounds are secreted in the fallopian tubes and probably help sperm in navigation towards the oocyte. Therefore some authors claim that female GAGs are also involved in sperm capacitation (Figure 1(b)). The mechanism may be thus more complex than previously thought and is still far from complete understanding. The ability to bind heparin and the other GAGs was also reported in human SP [46]. Like animal BSPs they are able to bind to the sperm surface via phosphatidylcholine. Lactoferrin seems to be a major sperm coating agent, but heparin binding ability was suggested to about 40 proteins, including precursors of MMP9, MMP2, PSA, hCG, semenogelin, and fibronectin. Their involvement in the capacitation of human sperm has not been reported. On the other hand, in humans capacitation control has been attributed to the unique glycoprotein, termed glycodelin. Glycodelin isoforms are synthesized in both male and female reproductive tracts. Only one isoform is present in the seminal plasma, designated as glycodelin S (Gd-S). The other three isoforms (Gd-A, Gd-C, and Gd-F) occur in different sites of the female reproductive tract. The appropriate sperm function involves sequential attachment of these isoforms, with Gd-S as the initial one. Interestingly, all glycodelin isoforms, whether produced in male or female reproductive organs, have an identical protein moiety. The difference among the isoforms concerns only the structure of oligosaccharides "decorating" protein surface, thus carbohydrates seem to be responsible for the sequential interactions with appropriate sperm surface receptors [47, 48]. At the moment of ejaculation Gd-S binds to its receptors, and in this form is able to traverse cervical mucus. After entering the uterine cavity, glycodelin S is shedded and this is the signal for sperm capacitation: albumin-induced remodeling of the cell membrane and cholesterol efflux (Figure 1(c)) [49–51]. Model studies have shown that the attachment of Gd-S inhibits cholesterol efflux, demonstrating the role of this protein as an agent preventing preterm capacitation. The female Gd-F and Gd-C isoforms are involved in initiation of acrosomal reaction and binding to* zona pellucida*. The last isoform, Gd-A, is induced immediately after embryo implantation, possibly providing protection as an immunosuppressive protein. Much attention has been devoted to the glycosylation of glycodelins, different in the male and female Gd glycoforms. It is worth noting that after deglycosylation the glycodelin isoforms lose their biological activity [49]. It could be thus suggested that inappropriate glycosylation may impair time and site regulation of sperm activation and thus reproductive functions. This hypothesis, however, still lacks solid experimental support.

## 4. Modulation of a Female Immune System

**Figure 2:**
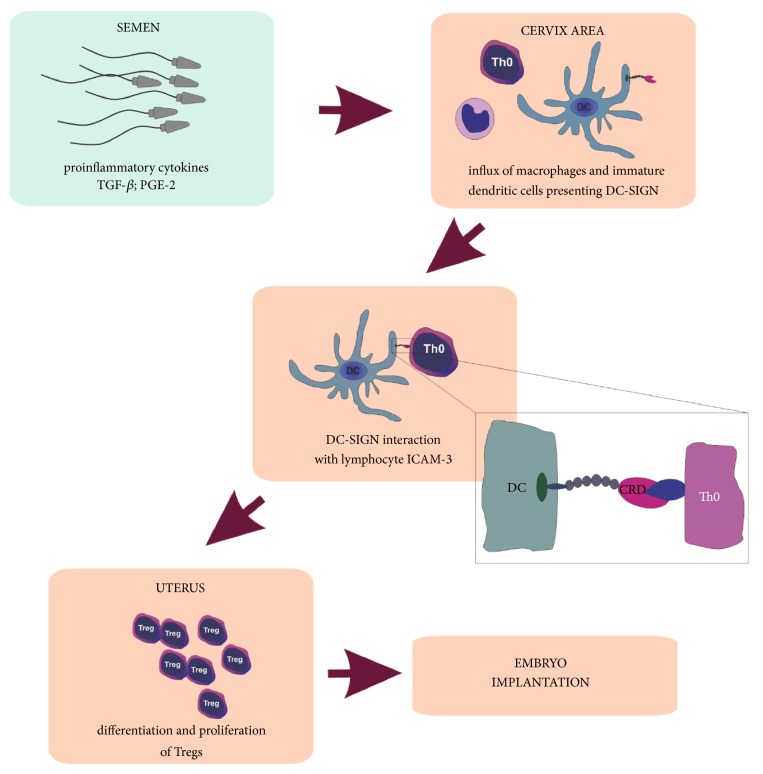
The hypothesized impact of seminal plasma components on the immunomodulation in female reproductive tract.

In the immune system the complexity and precision of antigenic recognition mechanisms enable effective fight against pathogens. In the context of fertilization, however, it becomes a great challenge. Over the last few decades, scientists have been trying to explain the one of the biggest puzzles in immunology, which is keeping male gametes and a fetus, presenting non-self-paternal antigens, safe from maternal immune reaction. The hypotheses that have been proposed to explain this phenomenon included antigenic immaturity of the fetus, strong suppression of mother's immune system during pregnancy, and the existence of impermeable placenta barrier. None of these hypotheses was fully true. Successful induction of the immune response by injection of mice fetal tissue in the skin graft tests proved antigen maturity of the fetus. Trophoblast cells penetrate and remodel the mother's spiral arteries to allow increased blood flow through the placenta [52], indicating a close contact of the mother to the fetus, thus excluding the concept of an absolutely sealed barrier [53]. Also, because of the risk of infection, the female immune system cannot be simply inactivated, especially since the vaginal environment is an important gate for pathogen invasion. Therefore, maternal immune system must reach an equilibrium that allows selective tolerance for gametes and developing trophoblast, without a decline in the effective response against pathogens. Moreover, despite the fact that spermatozoa exhibit numerous unique proteins, only a small percentage of women develop antisperm antibodies [54], which further confirms that sperm and seminal plasma are able to evade immune response in male and female reproductive systems. Deposition of the semen in the vagina induces an immediate postcoital leucocytic reaction [55]. In its course, the uterine epithelium is stimulated to synthesize proinflammatory cytokines, mainly IL-6, IL-1, IL-1*α*, and GM-CSF. Immunomodulatory factors that are supposed to trigger this reaction are abundant in the ejaculate [56, 57] (Figure 2). The result is massive infiltration of the cervix by monocytes, dendritic cells (DCs) and lymphocytes directly after coitus [58]. Especially DCs seem to be located in the center of the immune response associated with conception and early pregnancy [55]. The instruction from the DCs is a necessary signal for the proper launch and operation of the cellular response. However, dendritic cells are also able to weaken the reaction, and this ability places them as a key switch in homeostasis regulation. It is suggested that keeping DCs in a stage of incomplete maturation and later their differentiation towards the tolerogenic phenotype [59–62] are the key signals for T lymphocytes, which in turn transform into a regulatory population (Tregs), essential in the implantation period [4] (Figure 2).

Seminal plasma components that trigger the process include cytokines produced by Sertoli and Leydig cells in the testes and involved in the regulation of spermatogenesis. They can be also secreted by epididymides, prostate and seminal vesicles [16, 63, 64]. The semen of healthy, fertile men contains a broad range of immunomodulatory factors, as reported by Politch et al. [65]. These authors have analyzed more than 20 cytokines, chemokines and growth factors, differing in their level and frequency of detection, as shown in Table 1. This panel may be useful as a reference for further studies, focused on the possible alterations in the spectrum of immunomodulatory factors associated with decreased fertility. Such data are limited so far. Increased levels of proinflammatory IL-6 in SP of infertile subjects were reported independently by Seshardi et al. [66] and Camejo [67]. On the other hand, IL-10 level occurred elevated in the Seshardi et al. study, while it decreased in the work by Camejo. No significant differences were found in IL-11, IL-12, TNF*α*, and IFN*γ* [66]. However, both authors underline that cytokines work in a complex network and the effect of any single one cannot be considered reliable. The best studied cytokine in the semen is transforming growth factor *β* (TGF-*β*). The reported concentrations range from about 100 ng/mL [65] to as much as 500 ng/mL [26, 65, 68, 69], much more than observed in the other secretions. It occurs, however, that only 7% of TGF-*β* of seminal origin exists in an active form [70]. Though the acidic vaginal environment should be sufficient for at least partial activation, it does not happen due to the relatively high buffering capacity of the seminal plasma. The pH in the vagina increases from 4.3 to 7.2 in up to 8 seconds after ejaculation [71]. TGF-*β* is therefore activated by several seminal plasma enzymes, including serine proteases, tissue-type plasminogen activator and urokinase [72]. These enzymes are derived from accessory glands, distinct from those where TGF-*β* is synthesized. The cytokine contact with its activators is thus possible only after ejaculation. This mechanism seems to protect the male tissues from too high levels of active TGF- *β*. Together with prostaglandins, TGF- *β* is the main agent responsible for induction of the inflammatory response in the female reproductive tract, leading to the cervical recruitment of dendritic cells and their precursors - monocytes [73, 74]. TGF- *β* appears to be involved also in the next phase, attributed to immunosuppressive properties [56, 68, 69], and together with prostaglandin E2 (PGE2) strongly support further stages of the proliferation of mature Treg lymphocytes [75, 76] (Figure 2). The key role of TGF-*β* in this process was confirmed by experiments in a mouse model in which the exogenous supply of TGF-*β* to the embryos raised the vaginal number of Tregs and limited the risk of fetal loss. It has also been shown that anti-TGF-*β* antibodies were able to inhibit the immunosuppressive effect, which confirms the importance of this cytokine in modulation the immune response [77].

## 5. Prostaglandins as Immunomodulators

Comparing to the other body fluids, human seminal plasma contains high concentrations of prostaglandins, especially E series (PGE) [78]. PGE concentrations are several orders of magnitude higher than in serum, though individual variability is considerable [79]. The highest concentration was determined for PGE2, 19-hydroxyprostaglandin E1, and 19-hydroxyprostaglandin E2 [80, 81]. PGE2 is a potent mediator of immune function. It induces the transcription and translation of cyclooxygenase 2 (COX2) genes in vaginal epithelium, enhancing the further synthesis of eicosanoids from arachidonic acid [82]. The prostaglandin is responsible for the suppression of macrophages and neutrophils, and also limits the effector function and cytotoxic activity of NK and T cells. At the same time PGE2 activates dendritic cells, limiting their ability to attract naive, auxiliary and effector T lymphocytes and contributing to their differentiation to the tolerogenic phenotype [76]. A consequence is stimulation and proliferation of regulatory T lymphocytes, crucial in the achievement of maternal tolerance, involved in appropriate embryo implantation and development of the placenta [83]. This general mechanism is necessary to maintain efficacy in the selected immune responses needed in the normal functioning of the body and to suppress the response directed against neoantigens.

## 6. The Hypothesis of a Role of Carbohydrates in Fetoembryonic Defense

The hypothesis of the carbohydrate impact in the human fetoembryonic defense system was proposed in the 1990s. The concept assumes that protein-sugar interactions associated with the presence of specific glycoproteins in the human reproductive system are involved in the development of immune tolerance during fertilization and pregnancy. The sugar moiety of glycoproteins is supposed to modulate the immune response [28, 84, 85], and semen components play an important role in the development of this type of tolerance. The immunosuppressive effect is attributed to the particular glycosylation profile of the proteins. Evaluation of glycans within the male reproductive system revealed the presence of numerous oligosaccharide structures that are absent in serum glycoproteins of healthy humans. Sperm surface and seminal plasma of healthy, fertile men are rich in LewisX and LewisY antigens, glycans containing bisecting GlcNAc and also high mannose-type chains [86, 87]. These immunomodulatory oligosaccharides are potential ligands of endogenous lectins such as galectins or dendritic cells lectin DC-SIGN [28, 84, 86–89]. DC-SIGN is a type II membrane receptor containing an extracellular C-type lectin (carbohydrate recognition, CRD) domain, specific for mannose [88, 90]. It is expressed on the surface of dendritic cells before their complete maturation. DC-SIGN binding to ICAM-3 of naive T cells initiates the contact of these two cells. Inhibition of the binding by mannan confirms the importance of carbohydrate in the interaction [90]. Recent studies have shown that sugar specificity of DC-SIGN is much more complex than previously expected: apart from mannose, the lectin also recognizes fucosylated Lewis (X, Y, A, B, and sulfo-X) antigens. The DC-SIGN domains, located extracellularly between the cell membrane and the lectin domain, most probably participate in lectin polymerisation in the membrane and influence the regulation of sugar specificity. This in turn affects the interaction with other receptors involved in innate immunity, like TLR (Toll Like Receptors), which restrict the internalization and presentation of antigens [62]. These complex mechanisms are shown to be involved in the evasion of pathogens and tumor cells from the control of the immune system. Intracellular DC-SIGN domains modulate a pathway of protein synthesis, including cytokines. This modulation works differently depending on whether the lectin binds fucosylated or high mannose glycans. It is suggested that this distinction may be important for the direction of TLR signal to pro-(mannose) or anti-(Lewis antigens) inflammatory response [59, 60]. It seems that maternal tolerance for paternal antigens may be associated with a similar mechanism [28, 84, 85].

## 7. Conclusions

The reduced reproductive potential of men cannot be dedicated only to the reduced count, motility, or abnormal sperm morphology. It seems that also the structural studies of male gametes do not describe all the factors that can determine fertilization. Numerous components of the seminal plasma, the complex secretion of the glands of the male urogenital system, can have a significant effect on the proper activity of the sperm in their way to the oocyte. Seminal plasma factors are involved in time and site regulation of sperm capacitation. The changes observed in the immune response following the exposure of SP to female genital organs show that SP components play an important role in the regulation of maternal immunological tolerance, the factor necessary for successful fertilization, and embryo implantation. Explaining the molecular mechanisms of a complex cascade of processes leading to fertilization is still a challenge for researchers, and finding solutions may help many patients suffering from infertility.

## Figures and Tables

**Table 1:** Cytokine and chemokine detection in seminal plasma samples [Politch et al.].

Abundant in all analyzed samples	Frequent, low levels	Rare

TGF-*β*, IL7, IL8, SDF1*α*, MCP1	IL1, IL5, IL6, IL13, IL17, RANTES, MIP1*β*, IFN*α*, GCSF	MIP1, IL2, IL10, IL12, TNF*α*, IFN*γ*, GM-CSF

## References

[1] Boivin J., Bunting L., Collins J. A., Nygren K. G. (2007). International estimates of infertility prevalence and treatment-seeking: potential need and demand for infertility medical care. *Human Reproduction*.

[2] Esteves S. C., Miyaoka R., Agarwal A. (2011). An update on the clinical assessment of the infertile male. *Clinics*.

[3] Gilany K., Minai-Tehrani A., Savadi-Shiraz E., Rezadoost H., Lakpour N. (2015). Exploring the human seminal plasma proteome: An unexplored gold mine of biomarker for male Infertility and male reproduction disorder. *Journal of Reproduction and Infertility*.

[4] Lefièvre L., Bedu-Addo K., Conner S. J. (2007). Counting sperm does not add up any more: time for a new equation?. *Reproduction*.

[5] World Health Organization (2010). *WHO Laboratory Manual for The Examination And Processing of Human Semen*.

[6] Lewis S. E. (2007). Is sperm evaluation useful in predicting human fertility?. *Reproduction*.

[7] Nagler H. M. (2011). A solitary semen analysis can never predict normal fertility. *Nature Reviews Urology*.

[8] Wang C., Swerdloff R. S. (2014). Limitations of semen analysis as a test of male fertility and anticipated needs from newer tests. *Fertility and Sterility*.

[9] Hamada A., Esteves S. C., Agarwal A. (2011). Unexplained male infertility. *Human Andrology*.

[10] Hamada A., Esteves S. C., Nizza M., Agarwal A. (2012). Unexplained Male Infertility: Diagnosis and Management. *International Brazilian Journal of Urology*.

[11] Jeng H. A., Pan C., Chao M., Lin W. (2015). Sperm DNA oxidative damage and DNA adducts. *Mutation Research - Genetic Toxicology and Environmental Mutagenesis*.

[12] Pacey A. A. (2010). Environmental and lifestyle factors associated with sperm DNA damage. *Human Fertility*.

[13] Uppangala S., Pudakalakatti S., D’souza F. (2016). Influence of sperm DNA damage on human preimplantation embryo metabolism. *Reproductive Biology*.

[14] Havrylyuk A., Chopyak V., Nakonechnyyj A., Kurpisz M. (2015). Novel aspects of human infertility: the role of the male factor. *Postepy Higieny i Medycyny Doswiadczalnej*.

[15] Aitken R. J. (2016). Oxidative stress and the etiology of male infertility. *Journal of Assisted Reproduction and Genetics*.

[16] Fraczek M., Czernikiewicz A., Kurpisz M., Agarwal A., Aitken R. J., Alvarez J. G. (2012). Cytokines and oxidative stress in germ line. *Studies on Men Health and Fertility*.

[17] Sanocka D., Miesel R., Jedrzejczak P., Kurpisz M. K. (1996). Oxidative stress and male infertility. *Journal of Andrology*.

[18] Vatannejad A., Tavilani H., Sadeghi M. R., Amanpour S., Shapourizadeh S., Doosti M. (2017). Evaluation of ROS-TAC score and DNA damage in fertile normozoospermic and infertile asthenozoospermic males. *Urology Journal*.

[19] Walczak-Jedrzejowska R., Wolski J. K., Slowikowska-Hilczer J. (2013). The role of oxidative stress and antioxidants in male fertility. *Central European Journal of Urology*.

[20] Wagner H., Cheng J. W., Ko E. Y. (2019). Role of reactive oxygen species in male infertility: An updated review of literature. *Arab Journal of Urology*.

[21] Gharagozloo P., Aitken R. J. (2011). The role of sperm oxidative stress in male infertility and the significance of oral antioxidant therapy. *Human Reproduction*.

[22] Barazani Y., Katz B. F., Nagler H. M., Stember D. S. (2014). Lifestyle, environment, and male reproductive health. *Urologic Clinics of North America*.

[23] Silberstein T., Har-Vardi I., Harlev A. (2016). Antioxidants and polyphenols: concentrations and relation to male infertility and treatment success. *Oxidative Medicine and Cellular Longevity*.

[24] Kratz E. M., Kałuża A., Ferens-Sieczkowska M. (2016). Gelatinases and their tissue inhibitors are associated with oxidative stress: a potential set of markers connected with male infertility. *Reproduction, Fertility and Development*.

[25] Bedford J. M. (2015). The functions—or not—of seminal plasma?. *Biology of Reproduction*.

[26] Bromfield J. J. (2014). Seminal fluid and reproduction: much more than previously thought. *Journal of Assisted Reproduction and Genetics*.

[27] McGraw L. A., Suarez S. S., Wolfner M. F. (2015). On a matter of seminal importance. *BioEssays*.

[28] Clark G. F., Schust D. J. (2013). Manifestations of immune tolerance in the human female reproductive tract. *Frontiers in Immunology*.

[29] Schjenken J. E., Robertson S. A. (2014). Seminal fluid and immune adaptation for pregnancy—comparative biology in mammalian species. *Reproduction in Domestic Animals*.

[30] Baumgart E., Lenk S. V., Loening S. A., Jung K. (2002). Quantitative differences in matrix metalloproteinase (MMP)-2, but not in MMP-9, tissue inhibitor of metalloproteinase (TIMP)-1 or TIMP-2, in seminal plasma of normozoospermic and azoospermic patients. *Human Reproduction*.

[31] Caballero I., Parrilla I., Almiñana C. (2012). Seminal plasma proteins as modulators of the sperm function and their application in sperm biotechnologies. *Reproduction in Domestic Animals*.

[32] Silber S. J., Nagy Z., Liu J. (1995). Genetics: The use of epididymal and testicular spermatozoa for intracytoplasmic sperm injection: The genetic implications for male infertility. *Human Reproduction*.

[33] Grizard G., Chevalier V., Griveau J. F., Le Lannou D., Boucher D. (1999). Influence of seminal plasma on cryopreservation of human spermatozoa in a biological material-free medium: study of normal and low-duality semen. *International Journal of Andrology*.

[34] Ben W. X., Fu M. T., Mao L. K., Ming Z. W., Xiong W. W. (1997). Effects of various concentrations of native seminal plasma in cryoprotectant on viability of human sperm. *Systems Biology in Reproductive Medicine*.

[35] Donnelly E. T., McClure N., Lewis S. E. (2001). Cryopreservation of human semen and prepared sperm: effects on motility parameters and DNA integrity. *Fertility and Sterility*.

[36] de Graaf S., Leahy T., Marti J., Evans G., Maxwell W. (2008). Application of seminal plasma in sex-sorting and sperm cryopreservation. *Theriogenology*.

[37] Leahy T., de Graaf S. P. (2012). Seminal plasma and its effect on ruminant spermatozoa during processing. *Reproduction in Domestic Animals*.

[38] Maxwell W., de Graaf S., Ghaoui R., Evans G. (2007). Seminal plasma effects on sperm handling and female fertility. *Society for Reproduction and Fertility*.

[39] Rickard J. P., Pini T., Soleilhavoup C. (2014). Seminal plasma aids the survival and cervical transit of epididymal ram spermatozoa. *Reproduction*.

[40] Rickard J. P., Schmidt R. E., Maddison J. W. (2016). Variation in seminal plasma alters the ability of ram spermatozoa to survive cryopreservation. *Reproduction, Fertility and Development*.

[41] Soleilhavoup C., Tsikis G., Labas V. (2014). Ram seminal plasma proteome and its impact on liquid preservation of spermatozoa. *Journal of Proteomics*.

[42] Leahy T., Gadella B. M. (2011). Sperm surface changes and physiological consequences induced by sperm handling and storage. *Reproduction*.

[43] Monteiro G., Papa F., Zahn F. (2011). Cryopreservation and fertility of ejaculated and epididymal stallion sperm. *Animal Reproduction Science*.

[44] Tollner T. L., Bevins C. L., Cherr G. N. (2012). Multifunctional glycoprotein DEFB126—a curious story of defensin-clad spermatozoa. *Nature Reviews Urology*.

[45] Aitken R. J., Nixon B. (2013). Sperm capacitation: a distant landscape glimpsed but unexplored. *Molecular Human Reproduction*.

[46] Kumar V., Hassan M. I., Tomar A. K. (2009). Proteomic analysis of heparin-binding proteins from human seminal plasma: a step towards identification of molecular markers of male fertility. *Journal of Biosciences*.

[47] Koistinen H., Koistinen R., Dell A. (1996). Glycodelin from seminal plasma is a differentially glycosylated form of contraceptive glycodelin-A. *Molecular Human Reproduction*.

[48] Seppälä M., Koistinen H., Koistinen R., Chiu P. C. N., Yeung W. S. B. (2007). Glycosylation related actions of glycodelin: gamete, cumulus cell, immune cell and clinical associations. *Human Reproduction Update*.

[49] Chiu P. C., Chung M., Tsang H. (2005). Glycodelin-S in human seminal plasma reduces cholesterol efflux and inhibits capacitation of spermatozoa. *The Journal of Biological Chemistry*.

[50] Yeung W. S. B., Lee K.-F., Koistinen R., Koistinen H., Seppälä M., Chiu P. C. N. (2009). Effects of glycodelins on functional competence of spermatozoa. *Journal of Reproductive Immunology*.

[51] Yeung W. S., Lee K. F., Koistinen R. (2007). Glycodelin: a molecule with multi-functions on spermatozoa. *Society for Reproduction and Fertility*.

[52] Head J. R., Billingham R. E. (1986). Concerning the immunology of the uterus. *American Journal Of Reproductive Immunology*.

[53] Yule T. D., Montoya G. D., Russell L. D., Williams T. M., Tung K. S. K. (1988). Autoantigenic germ cells exist outside the blood testis barrier. *The Journal of Immunology*.

[54] Tung K. S., Teuscher C., Meng A. L. (1981). Autoimmunity to spermatozoa and the testis. *Immunological Reviews*.

[55] Adefuye A. O., Adeola H. A., Sales K. J., Katz A. A. (2016). Seminal fluid-mediated inflammation in physiology and pathology of the female reproductive tract. *Journal of Immunology Research*.

[56] Gorelik L., Flavell R. A. (2002). Transforming growth factor-beta in T-cell biology. *Nature Reviews Immunology*.

[57] Sharkey D. J., Tremellen K. P., Jasper M. J., Gemzell-Danielsson K., Robertson S. A. (2012). Seminal fluid induces leukocyte recruitment and cytokine and chemokine mRNA expression in the human cervix after coitus. *The Journal of Immunology*.

[58] Robertson S. A., Ingman W. V., O'Leary S., Sharkey D. J., Tremellen K. P. (2002). Transforming growth factor *β*—a mediator of immune deviation in seminal plasma. *Journal of Reproductive Immunology*.

[59] Garcia-Vallejo J. J., van Kooyk Y. (2013). The physiological role of DC-SIGN: A tale of mice and men. *Trends in Immunology*.

[60] García-Vallejo J. J., van Kooyk Y. (2009). Endogenous ligands for C-type lectin receptors: the true regulators of immune homeostasis. *Immunological Reviews*.

[61] van Liempt E., Bank C. M. C., Mehta P. (2006). Specificity of DC-SIGN for mannose- and fucose-containing glycans. *FEBS Letters*.

[62] Zhou T., Chen Y., Hao L., Zhang Y. (2006). DC-SIGN and immunoregulation. *Cellular & Molecular Immunology*.

[63] Fraczek M., Kurpisz M. (2015). Cytokines in the male reproductive tract and their role in infertility disorders. *Journal of Reproductive Immunology*.

[64] Maegawa M., Kamada M., Irahara M. (2002). A repertoire of cytokines in human seminal plasma. *Journal of Reproductive Immunology*.

[65] Politch J. A., Tucker L., Bowman F. P., Anderson D. J. (2007). Concentrations and significance of cytokines and other immunologic factors in semen of healthy fertile men. *Human Reproduction*.

[66] Seshadri S., Bates M., Vince G., Jones D. I. (2009). The role of cytokine expression in different subgroups of subfertile men. *American Journal of Reproductive Immunology*.

[67] Camejo M. (2009). Relation between immunosuppressive PGE 2 and IL-10 to pro-inflammatory IL-6 in seminal plasma of infertile and fertile men. *Systems Biology in Reproductive Medicine*.

[68] Nocera M., Chu T. M. (1993). Transforming growth factor beta as an immunosuppressive protein in human seminal plasma. *American Journal of Reproductive Immunology*.

[69] Robertson S. A. (2005). Seminal plasma and male factor signalling in the female reproductive tract. *Cell and Tissue Research*.

[70] Nocera M., Chu T. M. (1995). Characterization of latent transforming growth factor-*β* from human seminal plasma. *American Journal of Reproductive Immunology*.

[71] Suarez S. S., Pacey A. A. (2006). Sperm transport in the female reproductive tract. *Human Reproduction Update*.

[72] Chu T. M., Kawinski E. (1998). Plasmin, substilisin-like endoproteases, tissue plasminogen activator, and urokinase plasminogen activator are involved in activation of latent TGF-*β*1 in human seminal plasma. *Biochemical and Biophysical Research Communications*.

[73] Sharkey D. J., Macpherson A. M., Tremellen K. P., Mottershead D. G., Gilchrist R. B., Robertson S. A. (2012). TGF-*β* mediates proinflammatory seminal fluid signaling in human cervical epithelial cells. *The Journal of Immunology*.

[74] Sharkey D. J., Macpherson A. M., Tremellen K. P., Robertson S. A. (2007). Seminal plasma differentially regulates inflammatory cytokine gene expression in human cervical and vaginal epithelial cells. *Molecular Human Reproduction*.

[75] Ghiringhelli F., Puig P. E., Roux S. (2005). Tumor cells convert immature myeloid dendritic cells into TGF-*β*-secreting cells inducing CD4 +CD25 + regulatory T cell proliferation. *The Journal of Experimental Medicine*.

[76] Lenicov F. R., Rodrigues C. R., Sabatté J. (2012). Semen promotes the differentiation of tolerogenic dendritic cells. *The Journal of Immunology*.

[77] Ochsenkühn R., O’Connor A. E., Hirst J. J., Gordon Baker H., de Kretser D. M., Hedger M. P. (2006). The relationship between immunosuppressive activity and immunoregulatory cytokines in seminal plasma: Influence of sperm autoimmunity and seminal leukocytes. *Journal of Reproductive Immunology*.

[78] Samuelsson B. (1963). Isolation and identification of prostaglandins from human seminal plasma. 18. prostaglandins and related factors. *The Journal of Biological Chemistry*.

[79] Doncel G. F., Anderson S., Zalenskaya I. (2014). Role of semen in modulating the female genital tract microenvironment - implications for HIV transmission. *American Journal of Reproductive Immunology*.

[80] Kelly R. W., Carr G. G., Critchley H. O. (1997). A cytokine switch induced by human seminal plasma: an immune modulation with implications for sexually transmitted disease. *Human Reproduction*.

[81] Taylor P. L., Kelly R. W. (1974). 19-hydroxyiated E prostaglandins as the major prostaglandins of human semen. *Nature*.

[82] Joseph T., Zalenskaya I. A., Sawyer L. C., Chandra N., Doncel G. F. (2013). Seminal plasma induces prostaglandin-endoperoxide synthase (PTGS) 2 expression in immortalized human vaginal cells: involvement of semen prostaglandin E2 in PTGS2 upregulation. *Biology of Reproduction*.

[83] Aluvihare V. R., Kallikourdis M., Betz A. G. (2004). Regulatory T cells mediate maternal tolerance to the fetus. *Nature Immunology*.

[84] Clark G. F. (2014). The role of glycans in immune evasion: the human fetoembryonic defence system hypothesis revisited. *MHR: Basic science of reproductive medicine*.

[85] Clark G. F., Oehinger S., Patankar M. S. (1996). A role for glycoconjugates in human development: the human feto-embryonic defence system hypothesis. *Human Reproduction*.

[86] Pang P., Tissot B., Drobnis E. Z. (2007). Expression of bisecting type and lewis x/Lewis y terminated *N*-glycans on human sperm. *The Journal of Biological Chemistry*.

[87] Pang P.-C., Tissot B., Drobnis E. Z., Morris H. R., Dell A., Clark G. F. (2009). Analysis of the human seminal plasma glycome reveals the presence of immunomodulatory carbohydrate functional groups. *Journal of Proteome Research*.

[88] Geijtenbeek T. B. H., Torensma R., Van Vliet S. J. (2000). Identification of DC-SIGN, a novel dendritic cell-specific ICAM-3 receptor that supports primary immune responses. *Cell*.

[89] Pang P., Chiu P. C., Lee C. (2011). Human sperm binding is mediated by the sialyl-lewisx oligosaccharide on the zona pellucida. *Science*.

[90] Steinman R. M. (2000). DC-SIGN: a guide to some mysteries of dendritic cells. *Cell*.

